# Antiretroviral activity of 5-azacytidine during treatment of a HTLV-1 positive myelodysplastic syndrome with autoimmune manifestations

**DOI:** 10.1186/1743-422X-9-1

**Published:** 2012-01-03

**Authors:** Panagiotis T Diamantopoulos, Maria Michael, Olga Benopoulou, Efthymia Bazanis, George Tzeletas, John Meletis, George Vayopoulos, Nora-Athina Viniou

**Affiliations:** 1 1st Department of Internal Medicine, Haematology Unit, National and Kapodistrian University of Athens, "Laikon" General Hospital, Athens 11527, Greece

**Keywords:** HTLV-1, 5-azacytidine, Myelodysplastic syndrome, RAEB-1, Leukocytoclastic vasculitis, autoimmunity

## Abstract

Myelodysplastic syndromes (MDS) are often accompanied by autoimmune phenomena. The underlying mechanisms for these associations remain uncertain, although T cell activation seems to be important. Human T-lymphotropic virus (HTLV-1) has been detected in patients with myelodysplastic syndromes, mostly in regions of the world which are endemic for the virus, and where association of HTLV-1 with rheumatological manifestation is not rare. We present here the case of a 58 year old man who presented with cytopenias, leukocytoclastic vasculitis of the skin and glomerulopathy, and was diagnosed as MDS (refractory anemia with excess blasts - RAEB 1). The patient also tested positive for HTLV-1 by PCR. After 8 monthly cycles of 5-azacytidine he achieved a complete hematologic remission. Following treatment, a second PCR for HTLV-1 was carried out and found to be negative. This is the first report in the literature of a HTLV-1-positive MDS with severe autoimmune manifestations, which was treated with the hypomethylating factor 5-azacitidine, achieving cytogenetic remission with concomitant resolution of the autoimmune manifestations, as well as HTLV-1-PCR negativity. HTLV-1-PCR negativity may be due to either immune mediated clearance of the virus, or a potential antiretroviral effect of 5-azacytidine. 5-azacytidine is known for its antiretroviral effects, although there is no proof of its activity against HTLV-1 infection in vivo.

## Background

The association of myelodysplasia with autoimmune phenomena is well established for more than 20 years, as evidenced by several case reports and small patient series. HTLV-1, the first described human retrovirus, and cause of adult T cell leukemia/lymphoma (ATLL) has been associated to myelodysplastic syndromes. In areas endemic for the virus, serologic positivity for HTLV-1 is not very rare in patients with myelodysplastic syndromes [1]. Moreover there are several reports of autoimmune manifestations in HTLV-1 positive patients [2,3]. These correlations are very unusual in non-endemic areas, such as Greece.

## Case presentation

A 58 year old man was admitted to our clinic due to a 6-month history of night sweats, anorexia, and weight loss. 6 months ago he had an influenza-like illness and 4 months before admission, he developed ptosis of his left eyelid, with no other neurologic signs and symptoms and a negative brain MRI and carotid artery triplex ultrasound. The symptom resolved without any neurologic sequelae. One month before admission, he developed erythema nodosum on his left leg. His past medical history was unremarkable.

The physical examination did not reveal any abnormalities. He had no liver, spleen, or lymph node enlargement. No rash on the skin or mucosa was noted.

His complete blood count showed anemia, leucopenia and thrombocytopenia. He also had increased serum markers of inflammation, a marked hypergammaglobulinemia and a slightly affected renal and liver function along with a normal urinalysis Table 1. The evaluation included antibody testing for viral infections (HBV, HCV, HIV, HTLV, herpesviruses), Leishmania, Brucella, syphilis, Ricketsiae, and Toxoplasma. His immunologic profile showed low titers of antinuclear antibodies (1:80), high titers of Reumatoid Factor (250 IU/mL), and positive anti-citrullinated protein antibodies (17.7 U/mL, reference value < 15 U/mL), with complement levels (C_3_/C_4_/C_50_), antistreptolysin O titer, antimitochondrial andibodies, anti-smooth muscle antibodies, anti nutrophilic cytoplasmic antibodies, anti-b_2_-glycoprotein-I antibody, and anticardiolipin antibodies all being normal. Cryoglobulins were also negative, as was the direct Coombs test. The chest X-ray was normal, as was the heart ultrasound. A PPD (purified protein derivative) skin test was performed and found negative.

**Table 1:** Complete blood count with differential, and biochemical profile of the patient upon his first admission

Ht: 31.2%	Hb: 10.4 g/dL	MCV: 94.2 fl	RET: 0.84%	**PLT: 130 × 10^9^/L**.	
WBC: 2.6 × 10^9^/L	Differential: neutrophils 37%, lymphocytes: 53%, monocytes: 4%, eosinophils: 1%, basophils: 5%, with several neutrophils showing granulation disorders and small cytoplasmic vacuoles				

BUN: 33 mg/dL	Cr: 1.4 mg/dL	ALP: 399 U/L	γGT: 127 U/L	AST: 27 U/L	ALT: 66 U/L

ESR: 135 mm/1st hour	CRP: 60 mg/L				

He underwent an extensive imaging evaluation with a chest, abdominal and pelvic CT scan and a brain MRI, as well as a percutaneous liver biopsy that revealed a mild steatohepatitis.

**Figure 1:**
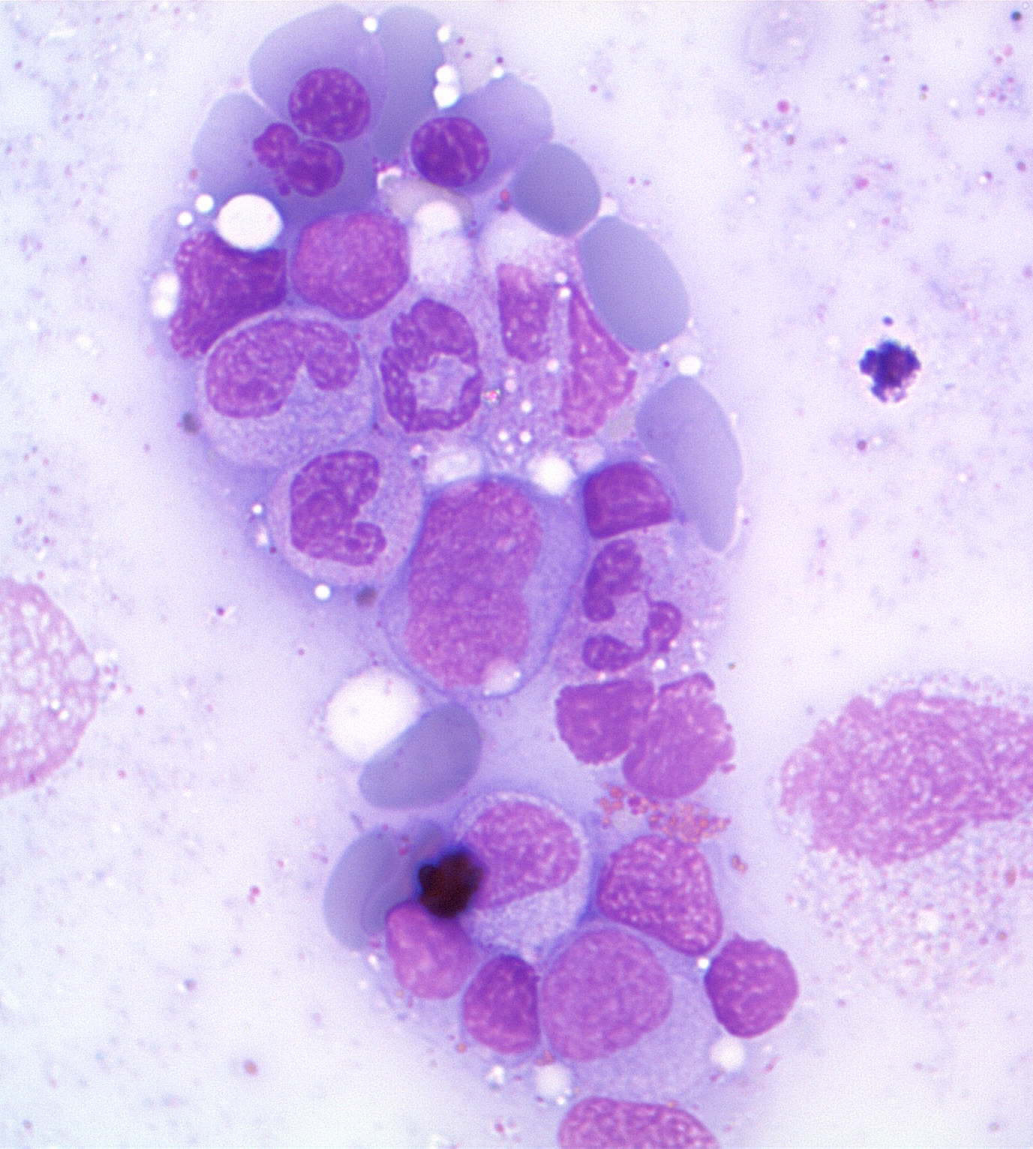
**Patient's bone marrow smears showing dysplastic changes of erythroid cells and immature/dysplastic forms of myeloid cells**.

**Figure 2:**
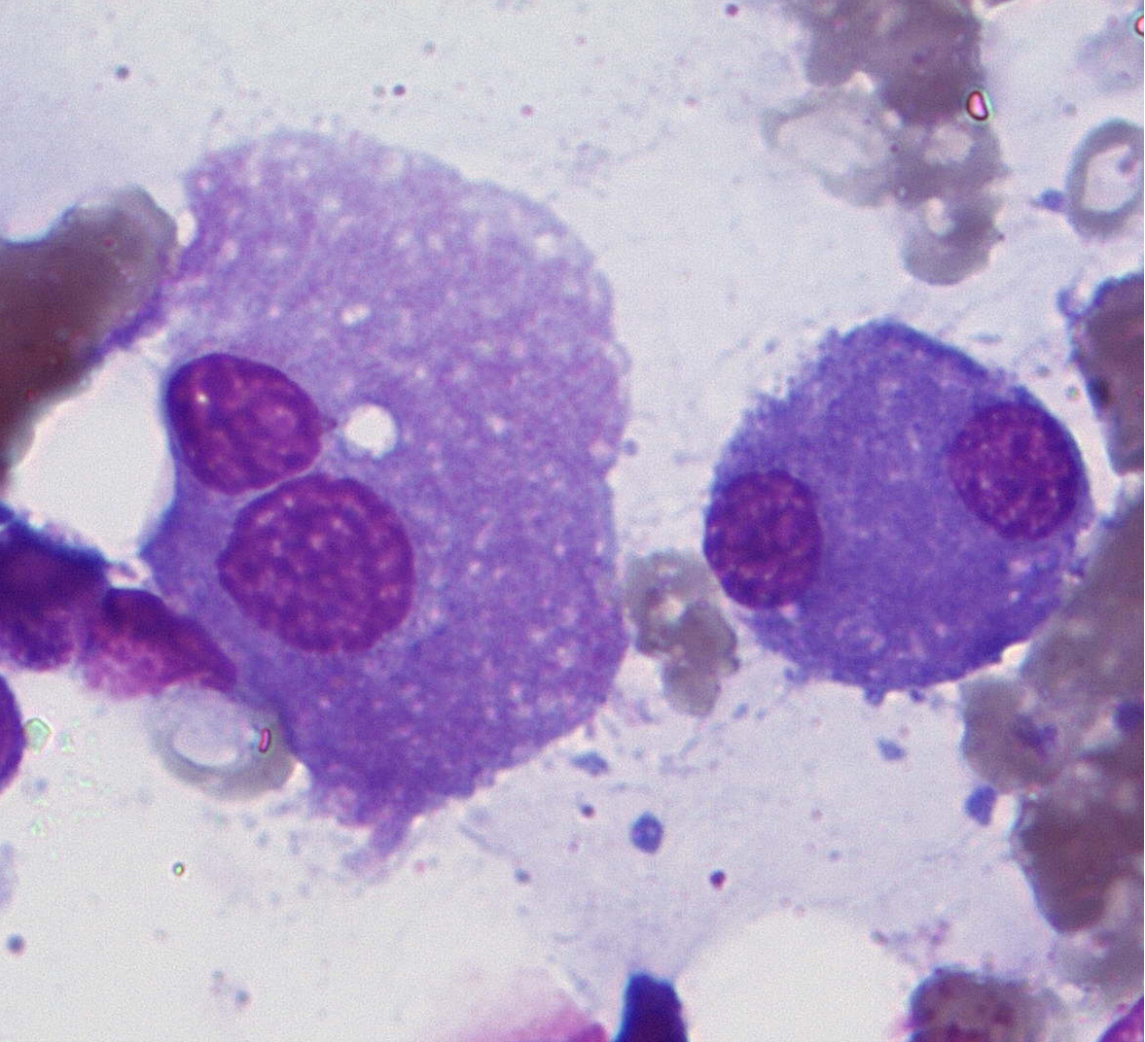
**Typical dysplastic megacaryocytes**.

Finally, he had a bone marrow aspiration and biopsy that confirmed the diagnosis of myelodysplastic syndrome with 8% blasts (RAEB-1, WHO 2008) [4] (Figures 1 and 2), while immunophenotypic analysis revealed the myeloid origin of the blasts. Karyotypic analysis of the bone marrow showed a pseudo-hypodiploid abnormal clone of 45, X-Y in 24 out of 25 studied metaphases. The patient also tested positive for HTLV-1 with a diagnostic PCR, both in blood and bone marrow. The result was confirmed in a second sample. A T-cell receptor gene clonality assay was carried out and a clonal population of T cells was revealed.

During his hospitalization he presented fever, a vasculitic rash at his neck, torso and arms (histopathologic examination showed a leukocytoclastic vasculitis), along with deterioration of his anemia (Ht = 26%), thrombocytopenia (20 × 10^9^/L), liver (ALP 1216 U/L, SGOT 61 U/L, SGPT 124 U/L) and renal function (Creatinine 1.6 mg/dL), and an abnormal urine sediment (1400 mg urine protein per day, 40-50 red blood cells per high power field [70% of them being of glomerular origin] and granular casts). A renal biopsy was scheduled in order to evaluate his nephritic syndrome, but was cancelled due to severe thrombocytopenia with positive antiplatelet antibodies.

After an unsuccessful course of antibiotics, in an effort to control fever, rash and glomerulopathy, methylprednisolone was administered (40 mg bis) resulting in rapid improvement of his clinical findings and biochemical profile. Methotrexate 10 mg/week PO was added to the regimen after 2 unsuccessful attempts of steroid tapering, while, due to persistent and severe pancytopenia, he was treated with a course 8 monthly cycles of 5-azacytidine in a dose of 75 mg/m^2^/d for 7 days. He achieved complete hematologic remission that was further confirmed via immunophenotypic analysis.

Fifteen months after initial diagnosis, bone marrow karyotypic and molecular testing (MLPA and QF-PCR for several chromosomal regions, including 2p, 5q, 6q25-26, 7p12.2, 8q24.21, 9p13.2, 9p21.3, 10q23, 11q22.3, 12p13.2, 13q14, 17p13.1, and 21q22.1, and for JAK2 V617F mutation) revealed no abnormalities. About 15 months after the first positive PCR for HTLV-1, and 11 months after initiation of azacytidine, a second test was carried out and was found negative.

One month later, the patient underwent allogeneic peripheral blood stem cell transplantation from a fully matched unrelated donor with a once daily intravenous busulfan and fludarabine myeloablative conditioning regimen. A bone marrow aspirate on day +30 demonstrated trilineage engraftment and complete chimera (100% donor). On day +58 the patient developed grade II graft versus host disease (GVHD) of the skin that responded promptly to treatment with steroids. Currently, 8 months after transplantation, the patient remains in complete remission.

## Discussion

The association of myelodysplasia with autoimmunity is well established for almost 20 years and has been confirmed by clinical and laboratory data [5,6].

There are several reports in the literature correlating various autoimmune manifestations such as leukocytoclastic vasculitis [7], relapsing polychondritis [8], polyarteritis nodosa, Grave's disease, Sjögren's syndrome [9], Wegener's granulomatosis [10], bronchiolitis obliterans [11], autoimmune hemolytic anemia [12], and thrombocytopenia [13,14], red cell aplasia [15], dermatomyositis [16], peripheral polyneuropathy and inflammatory bowel disease [17], giant cell arteritis/polymyalgia rheumatica [18,19], and Addison's disease [20].

MDSs have been linked to several types of glomerular diseases, such as glomerulosclerosis, membranous nephropathy, IgA nephropathy, and ANCA associated nephropathy [21-25]. Unfortunately, in our case, due to lack of histopathologic examination, only speculations can be made about the type of the patient's renal disease.

Both the innate and adoptive immune system have been implicated in the autoimmune manifestations of patients with MDS in several studies evaluating T cell activation, function, and kinetics, as well as associations with the HLA system [26,27].

A large number of reports favor the use of immunoregulatory treatments [28] such as antithymocyte globuline, cyclosporine, thalidomide, steroids, mycophenolate mofetil, anti-TNF, and methotrexate in patients with MDS and autoimmune manifestations. More recently, guidelines for the use of immunomodulatory treatment for MDS have been proposed [29].

In our case, myelodysplastic syndrome and autoimmune phenomena were also accompanied by a positive PCR for HTLV-1. HTLV-1 has been implicated in the pathogenesis of ATLL and tropical spastic paraparesis. Although the virus is oncogenic, it neither induces oncogenesis by encoding oncogenes, nor does it integrate into the host genome to disrupt host gene expression. Rather, viral gene products interact with host proteins (mainly transcription factors) altering their function.

It is estimated that 10-20 million people are infected by the virus worldwide. In endemic areas (southern Japan, the Caribbean, South America, the Melanesian islands, Papua New Guinea, the Middle East and central and southern Africa) seroprevalence ranges from 3% to 30%. In non-endemic areas, such as Greece, seroprevalence is less than 1%. Seropositivity among blood donors in Greece was reported to be 0.009% in a large multicenter study [30].

Transmission of the virus is mainly achieved through breast feeding, sexual intercourse, inappropriate sterilization procedures and blood transfusion or injection drug use. Our patient claimed not to be an injection drug user, and had never been transfused in the past. His wife tested negative for HTLV-1.

In the literature serologic positivity for the virus has been detected in patients with myelodysplastic syndromes in both endemic [31] and non endemic regions for the virus [32].

Most studies about the prevalence of HTLV-1 in hematologic diseases come from Japan where the infection is endemic. Several case reports suggest the possible association between immune thrombocytopenia (ITP) and HTLV-1 infection [33]. Recently Inoue et al. studied HTLV-1 prevalence in patients with RAEB/RAEBt and AML [31]. The high prevalence (28.3%) of HTLV-1 infection in the studied group of patients with RAEB/RAEBt is very interesting but cannot be extrapolated to patients of non-endemic regions. There is only one study in the literature coming from a non endemic region that suggests a possible role of HTLV-1 infection in the pathogenesis of hematologic diseases other than ATLL, especially MDS [32].

Association of the virus with rheumatologic manifestations is not rare and includes uveitis, chronic inflammatory arthropathy, Sjögren's syndrome, lymphocytic alveolitis, polymyositis, and fibromyalgia, but all these reports come from regions endemic for the HTLV-1 infection [33-40]. Moreover, there are 3 reports, coming from endemic regions, associating HTLV-1 with glomerulonephritis and interstitial nephritis [35,41,42].

Treatment of the diseases caused by HTLV-1 is problematic. There have been only limited studies of specific antiretroviral therapy using nucleoside analogue reverse transcriptase inhibitors for HTLV-1 infection [43-45].

Azacytidine and its deoxy derivative, decitabine (5-aza-2'deoxycytidine), are FDA-approved agents for the treatment of low and high risk patients with MDS. Azacytidine is incorporated into DNA and RNA by methyltransferases and acts as a false substrate and a potent inhibitor of methyltransferases. This results in reduction of DNA methylation (hypomethylating or demethylating agent) affecting the binding of cell regulation proteins to the DNA/RNA substrate [46,47]. DNA hypermethylation at the CpG islands has been described in MDS. These CpG islands are targets for transformation-associated aberrant hypermethylation activity during leukemogenesis. Moreover, aberrant CpG island hypermethylation seems to be an important multistep process in the development of ATLL [48].

It has been shown that 5-azacytidine inhibits human immunodeficiency virus type 1 (HIV-1) replication [49]. This antiviral activity can be attributed to an increase in the frequency of viral mutants, achieved by incorporation of its derivative 5-aza-2'-deoxycytidine into the viral DNA. The same may apply to other retroviruses such as HTLV-1, for which it has been shown that its transcriptional activity is regulated by methylation [50]. Recently, a closely related molecule, decitabine, has been successfully used in a trial to reduce HIV infectivity [51]. The antiviral activity of the drug has been attributed to an increase in the mutational load that inhibits the generation of infectious progeny virus from provirus.

The antiretroviral activities of hypomethylating factors, such as decitabine and 5-azacytidin are the subject of several current studies. One of them suggests a more specific role of 5-azacytidine in the treatment of ATLL, as it shows a growth inhibition of leukemic cells, offering a potential new therapeutic approach to improve the poor outcomes associated with ATLL [52].

Although in our case, HTLV-1 negativity may have resulted from other unidentified factors, such as immune restoration following treatment, our case may be another example of drug repositioning, were azacitidine administered to treat MDS, had also an effect on HTLV-1 replication, resulting into a negative PCR for the virus several months following treatment.

## Conclusions

This is a unique case of a patient with RAEB-1, accompanied by severe autoimmune manifestations, that was infected with HTLV-1 in a region non-endemic for the virus. The patient achieved a complete remission of his hematologic disease and autoimmune manifestations after treatment with 5-azacytidine, while several months later he tested negative for HTLV-1 by nested PCR, a fact that can be linked to the, already identified by other studies, antiretroviral action of the drug.

## Consent

Written informed consent was obtained from the patient for publication of this Case Report.

## Competing interests

The authors declare that they have no competing interests.

## Authors' contributions

PTD, Acquisition analysis and interpretation of data, drafting of the manuscript and revising the manuscript. MM, Acquisition and analysis of data. OB, Revising the manuscript. EB, Acquisition and analysis of data. GT, Acquisition and analysis of data. JM, Revision of the manuscript. GV, Revision of the manuscript. NAV, Final revision and approval of the manuscript.
